# HIV-1 and BLV are insensitive to SERINC5 restriction under the cell-cell infection

**DOI:** 10.1128/spectrum.02748-24

**Published:** 2025-01-27

**Authors:** Changqing Yu, Faming Jiang, Yujing Li, Qiushui Li, Kenzo Tokunaga, Dan Yang, Chao Xu, Nan Li, Sunan Li, Ilyas Khan, Yuanhua Xian, Changyou Xia, He Zhang

**Affiliations:** 1 Engineering Center of Agricultural Biosafety Assessment and Biotechnology, School of Advanced Agricultural Sciences, Yibin Vocational and Technical College, Yibin, China; 2 State Key Laboratory for Animal Disease Control and Prevention, Harbin Veterinary Research Institute, Chinese Academy of Agricultural Sciences, Harbin, China; 3 College of Veterinary Medicine, Sichuan Agricultural University, Chengdu, China; 4 Department of Pathology, National Institute of Infectious Diseases, Tokyo, Japan; 5 Key Laboratory of Climate, Resources and Environment in Continental Shelf Sea and Deep Sea of Department of Education of Guangdong Province, Department of Oceanography, Key Laboratory for Coastal Ocean Variation and Disaster Prediction, College of Ocean and Meteorology, Guangdong Ocean University, Zhanjiang, China; University of Arkansas Fayetteville, Fayetteville, Arkansas, USA

**Keywords:** serine incorporator 5, retrovirus, HIV-1, BLV, cell-cell, restriction

## Abstract

Serine incorporator 5 (SERINC5, SER5) suppresses viral cell-free infection. However, its antiviral potency under viral cell-cell infection is not examined yet. Here, we established the *in vitro* cell-cell infection systems to assess SER5’s antiviral activity on HIV-1 and bovine leukemia virus (BLV). Our results showed SER5 from different mammalian species, including *Homo sapiens*, *Bos taurus*, and *Felis catus*, was capable of significantly inhibiting HIV-1 cell-free infection. However, these SER5s were unable to restrict HIV-1 cell-cell infection. Intriguingly, Ebola virus glycoprotein-mediated pseudoviral cell-free infection was greatly enhanced by *Homo sapiens* and *Bos taurus* SER5. Notably, BLV was also insensitive to SER5-mediated restriction under the cell-cell infection. In addition, BLV envelope glycoproteins, which contained one glycosylation variation, were not restricted by SER5 in the model of cell-cell infection. Overall, our results indicated SER5 was ineffective against HIV-1 and BLV under the cell-cell infection, which was utilized by them to circumvent SER5-imposed restriction *in vitro* and may aid their transmission *in vivo*.

**IMPORTANCE:**

SER5 potently inhibits virus infection under the cell-free mode. However, few studies check whether SER5 keeps its restriction under the virus cell-cell transmission. In this work, we uncover SER5 loses its restriction to HIV-1 and BLV under the mode of cell-cell infection, demonstrating the viruses could employ this mode to overcome SER5 restriction and thus facilitate their transmission.

## INTRODUCTION

Generally, viruses employ two typical modes to spread in host cells, namely, the cell-free and cell-cell transmission. Lentiviruses like HIV-1 gain intercellular transmission through both styles. Whereas for the *deltaretrovirus genus* bovine leukemia virus (BLV) and human T-lymphotropic virus, their intercellular spread is strictly dependent on the cell-cell transmission via the virological synapses (1). Indeed, the cell-cell transmission helps viruses alleviate the pressure imposed by host-neutralizing antibodies and evade host innate immunity factors’ restriction. In viral infection cycles, many host membrane-associated antiviral factors could block their replication, such as bone marrow stromal antigen 2 (BST-2), serine incorporator 5 (SERINC5, SER5), membrane-associated RING-CH protein 8 (MARCH8), and interferon-induced transmembrane protein 3 (2–5). BST-2 is located at the cell membrane, where it tethers virus budding. Viral genes like HIV-1 vpu and simian immunodeficiency virus nef/env could downregulate BST-2 cell surface display (6, 7) and enhance viral cell-free infection. Alternatively, the virus utilizes the cell-cell transmission to overcome BST-2 restriction (8). Similar to BST-2, SER5 is also located at the cell membrane and identified to potently block retrovirus infection. SER5 is incorporated into virions when they bud from producer cells. In the next round of infection, SER5 is believed to impair viral envelope fusion with cell plasma membrane and thus block the virus infection (3, 5). Correspondingly, viruses evolve some genes to overcome SER5-imposed restriction. Nef, glycoGag, and S2 from HIV-1, murine leukemia virus, and equine infectious anemia virus (EIAV) (9, 10), respectively, could efficiently counteract SER5’s antiviral potency by downregulating its cell surface presentation. The SER5 activity is also regulated by the host E3 ligase and kinase (11, 12). Interestingly, it was found SER5 could diminish hepatitis B virus infection through lowering its production (13). However, these current studies are mainly focused on exploring SER5 antiviral activity in the cell-free infection and less is known about its role under the viral cell-cell infection. Here, we utilize the cell-cell infection system to assess SER5 antiviral potency on HIV-1 and BLV. Our results indicate SER5 is unable to suppress HIV-1 and BLV cell-cell infection, providing a new clue about how viruses evade SER5’s suppression.

## MATERIALS AND METHODS

### Phylogenetic tree construction

The Megalign program was applied to conduct SER5 sequence alignment. The phylogenetic tree was made by MEGA6 via the maximum likelihood method and the Hasegawa-Kishino-Yano model. The bootstrap value was based on 1,000 replicates. Phylogenetic analysis was performed with GenBank-deposited sequences, including XM_027552960.1 (*Bos taurus*), XM_012180719.3 (*Ovis aries*), XM_013994946.2 (*Sus scrofa*), XM_023618093.1 (*Equus caballus*), XM_011284262.3 (*Felis catus*), XM_536311.5 (*Canis lupus familiaris*), NM_172588.2 (*Mus musculus*), NM_001174072.3 (*Homo sapiens*), XM_015140335.2 (*Macaca mulatta*), and XM_002940195.5 (*Xenopus tropicalis*).

### Plasmids

The HIV Env-deficient proviral vector pNL-ΔEnv, HIV-Luc reporter proviral vector pNL-ΔEnv-luc, HIV-1 Env expression vector pNLnΔBS, human SER5-FLAG, SER5-GFP, MARCH8-GFP, and the N-terminal FLAG-tagged Ebola virus glycoprotein (EBOV GP)-ΔMLD expression vectors were used as previously described (14–18). The HIV-1 Gag-deficient vector pNL-ΔGag-Env was constructed via deletion of partial gag gene of the pNL-4.3 plasmid by the homologous recombination kit (Vazyme Biotech, Nanjing, China). Bovine and feline SER5s were acquired from peripheral blood mononuclear cells by Reverse Transcription-Polymerase Chain Reaction (RT-PCR) amplification with a C-terminal FLAG-tag and cloned into pcDNA3.1(+) and pEGFP-N1 vectors by KpnI and AgeI restriction sites, generating bovine and feline SER5-FLAG and SER5-GFP expression vectors. The BLV cDNA infectious clone BLV344 and the pSG-Env expression vector were provided by Luc Williems (University of Liege, Belgium). The two N-glycosylation site mutants (N230E and N251E) were performed by site-directed mutagenesis, based on the pSG-Env vector backbone.

### Cell lines

Human embryonic kidney-293T (293T) cells and Vero cells were acquired from American Type Culture Collection (ATCC). TZM-Bl, F81, and HeLa cells were purchased from Type Culture Collection Centre of the Chinese Academy of Sciences (Shanghai, China). All cells were maintained in 10% fetal bovine serum and cultured at 37°C with humidified 5% CO_2_.

### Confocal microscopy assay

HeLa cells (2 × 10^5^) were seeded on glass slides 10 h before transfection and transfected with Lipofectamine 3000 reagent (Thermo Fisher). Thirty-six hours later, the cells were washed, fixed with 4% paraformaldehyde, and permeabilized with 0.1% Triton X-100. After washing, the cells were blocked with 5% bovine serum albumin for 30 min. Following three washes with PBS, cells were stained with DAPI for 10 min, washed for 1 h at room temperature (RT), and used for confocal microscopy observation (ZEISS, LSM880).

### Western blotting assay

293T cells (5 × 10^5^) were seeded on six-well plates 12 h before transfection. Forty-eight hours post-transfection, the cells were lysed with radioimmunoprecipitation assay (RIPA) buffer (Sigma) and cleared by centrifugation. The supernatants were collected, mixed gently with loading dye (no boiling), and directly subjected to SDS-PAGE assay. The protein samples were then transferred to the polyvinylidene difluoride membrane, which was blocked with 4% nonfat milk for 1 h at RT. The membrane was then incubated with the primary antibody for 1 h at RT. After washing for 30 min, the membrane was subjected to a horseradish peroxidase (HRP)-conjugated secondary antibody incubation for 30 min at RT. Following washes for 30 min, the membrane was used for enhanced chemiluminescence (ECL) exposure (Thermo Fisher).

### Antibodies

Mouse anti-β actin and mouse anti-HA monoclonal antibodies were purchased from Sigma. Mouse anti-HIV P24/P55 monoclonal antibody was purchased from Sino Biology (China). HRP-conjugated anti-mouse immunoglobulin G secondary antibodies were purchased from Pierce.

### Viral infectivity assay

For HIV-1 Env/EBOV GP-mediated pseudoviral cell-free assay, a total of 5 × 10^5^ 293T cells were seeded on six-well plates 12 h before transfection. 293T cells were cotransfected with 0.5 µg pNLnΔBS (HIV-1 Env)/GPΔMLD (EBOV GP), 1.5 µg pNL-ΔEnv/pNL-ΔEnv-luc (viral core), and 0.5 µg SER5-FLAG expression vectors. Forty-eight hours post-transfection, virion-containing supernatants were collected and subjected to ultracentrifugation at 40,000 × *g* for 1 h (Beckman). Pellets were collected and used for the virion component western blotting (WB) assay. Simultaneously, 100 µL of each supernatant was used for P24^Gag^ quantity normalization (TransGen Biotech, China). Supernatants with equal amounts of P24 were used to infect the indicator TZM-Bl/Vero cells. Thirty-six hours later, viral infectivity was assessed via luciferase activity assay. For HIV-1 cell-cell infection assay, a total of 2 × 10^5^ 293T cells were seeded on 12-well plates 12 h before transfection. 293T cells were cotransfected with 0.4 µg pNL-ΔGag-Env and 1.2 µg SER5-FLAG expression vectors. After 24 h, cells were collected for co-culture with TZM-Bl cells. Before co-culture with 293T cells, 4 × 10^4^ TZM-Bl cells were seeded on 24-well plates. After 12 h, 293T cells were added to TZM-Bl cells. After 24 h, cells were lysed and used for luciferase activity assay.

### Luciferase activity assay

After 36 h post-infection, cell culture was terminated. Cells were washed three times with PBS and lysed with 100 µL of RIPA buffer. A total of 25 µL of cell lysate was taken, mixed with an equal volume of substrate (Bright-Glo Luciferase Assay System, Promega) for 10 min at RT, and then used for luminescence activity measurement.

### Syncytium formation assay

A total of 1 × 10^5^ F81 cells were seeded on 24-well plates 1 day before syncytium formation (SF) assay. 293T cells (1 × 10^5^) were transfected with 0.25 µg BLV344/pSG-env expression vector and 0.5 µg SER5-FLAG expression vector in 24-well plates. One day later, cells were digested and added to F81 cells with a ratio of 1:8 (293T cell: F81 cell). After 24 h, cells were fixed with 4% paraformaldehyde and incubated with the Wright’s stain reagent (Solarbio, China). Then, SF was checked, numerated (more than 20 nuclei were scored), and pictured under microscopy.

### Statistical analysis

The statistical analysis was performed by the GraphPad Prism program via one-way ANOVA and paired two-tailed Student’s *t*-test. *P* < 0.05 was considered as significant.

## RESULTS

### SER5 sequence alignment and evolutionary analysis

**Fig 1:**
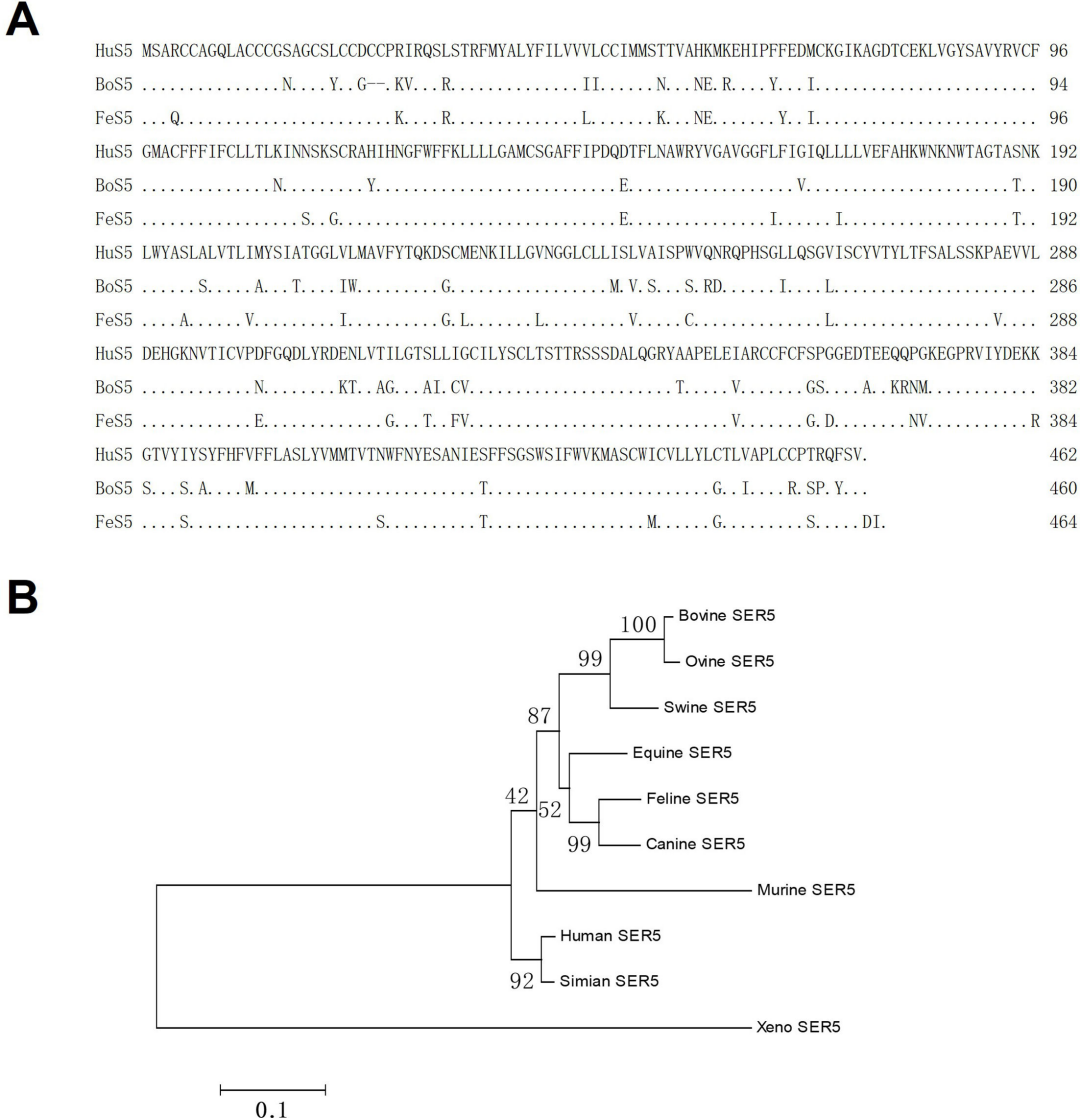
SER5 sequence alignment and evolutionary analysis. (**A**) SER5 amino acid (AA) sequences were aligned among *Homo sapiens* (HuS5), *Bos taurus* (BoS5), and *Felis catus* (FeS5). BoS5 and FeS5 AA identical to HuS5 were indicated as black dots. Horizontal line meant AA deletion. (**B**) SER5 sequences from different species were used for phylogenetic tree construction. HuS5, human SER5; BoS5, bovine SER5; FeS5, feline SER5.

In this report, we cloned bovine SER5 (BoS5) and feline SER5 (FeS5) and made an alignment with human SER5 (HuS5). Sequence analysis showed a more than 85% amino acid identity among these SER5s (Fig. 1A), which should reflect their conformational similarity. The variation sites were evenly distributed within the whole SER5 sequence. Evolutionary analysis showed that BoS5 had a far distance from HuS5, as compared with FeS5 (Fig. 1B). Overall, these results confirmed a highly conserved feature of SER5 among different mammalian species, which argued for their functional similarity.

### SER5 subcellular localization and its incorporation into virions

**Fig 2:**
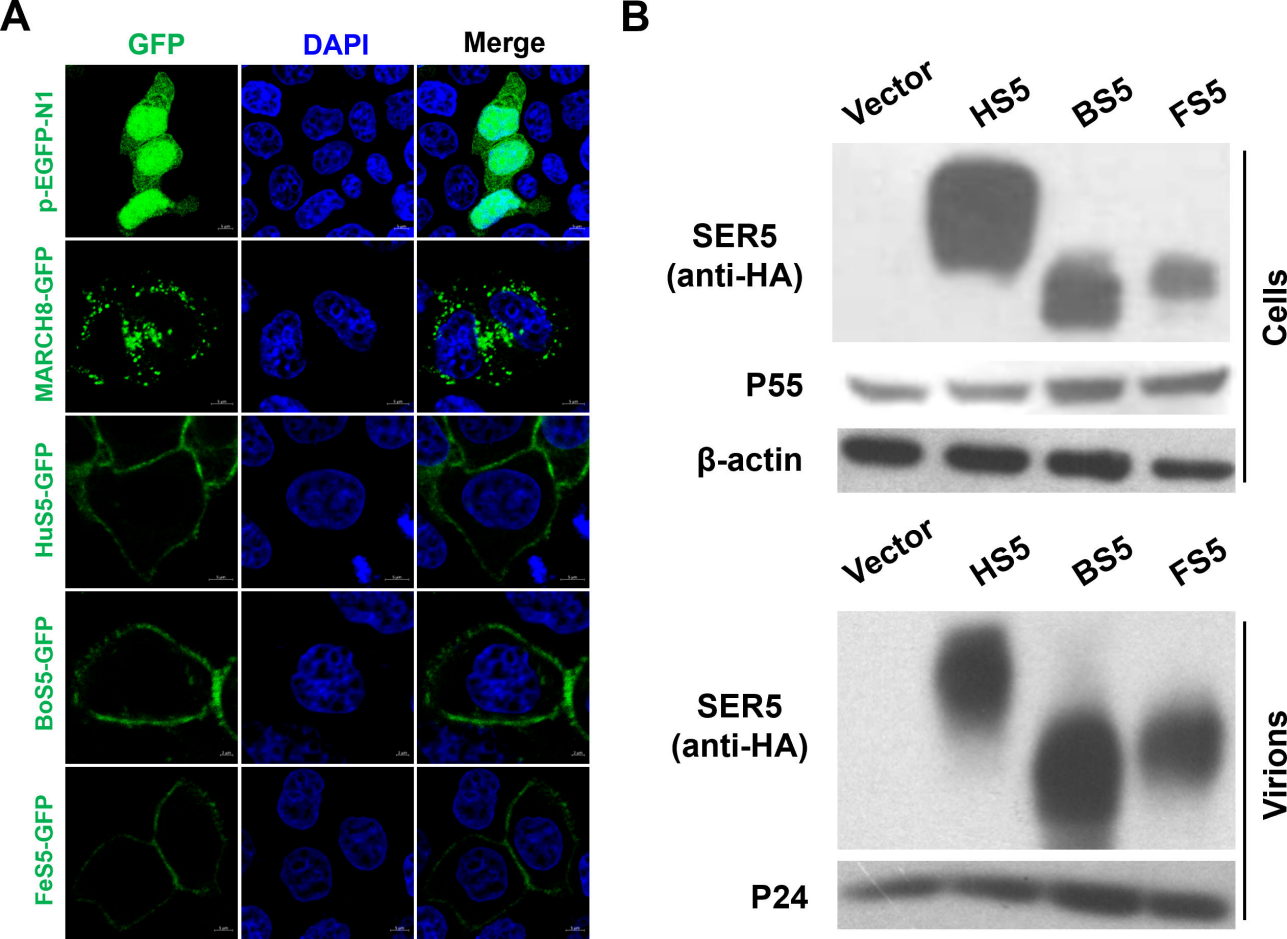
SER5 subcellular localization and its virion incorporation. (**A**) HuS5, BoS5, and FeS5 with a C-terminal GFP fusion were checked under the confocal microscopy. The p-EGFP-N1 and MARCH8-GFP vectors served as control. (**B**) HIV-1 viral core was packaged with HIV-1 envelope. Incorporation of HuS5, BoS5, and FeS5 into HIV-1 pseudovirions was detected via WB assay.

SER5 was previously reported to reside on the cell membrane, where it was packaged into the budding virions. Cell surface localization of SER5 was related to its antiviral efficacy. Thus, to investigate the antiviral role of BoS5 and FeS5, we initially determined their subcellular localization. To perform this work, a GFP-tag was fused in the C-terminus of BoS5, FeS5, and HuS5. As was shown in Fig. 2A, the GFP control diffused throughout the cytoplasm, whereas MARCH8-GFP showed a scattered punctate distribution, as previously showed (17). Distinct from these two molecules, HuS5-GFP displayed an obviously membrane-associated localization, which was consistent with the previous report (16). Similar to HuS5-GFP, BoS5-GFP and FeS5-GFP also showed a clearly membrane-associated feature, indicating their cell surface localization. Next, we examined whether these SER5s were incorporated into virions. Here, we applied a HIV-1 Env pseudotyped viral system. Briefly, 293T cells were co-transfected with the HIV-1 Env-deficient viral core, HIV-1 Env expression vector, and the SER5 expression vectors. Forty-eight hours later, pseudovirion-containing supernatants were collected and subjected to ultra-centrifugation. As shown in Fig. 2B, similar to HuS5, BoS5 and FeS5 could be potently packaged into the HIV-1 Env-based pseudovirions, which was in accordance with previous studies (3, 5, 16) and proved the necessity of virion incorporation for SER5’s antiviral potency.

### Distinct role of SER5 on HIV-1 cell-free and cell-cell infection

**Fig 3:**
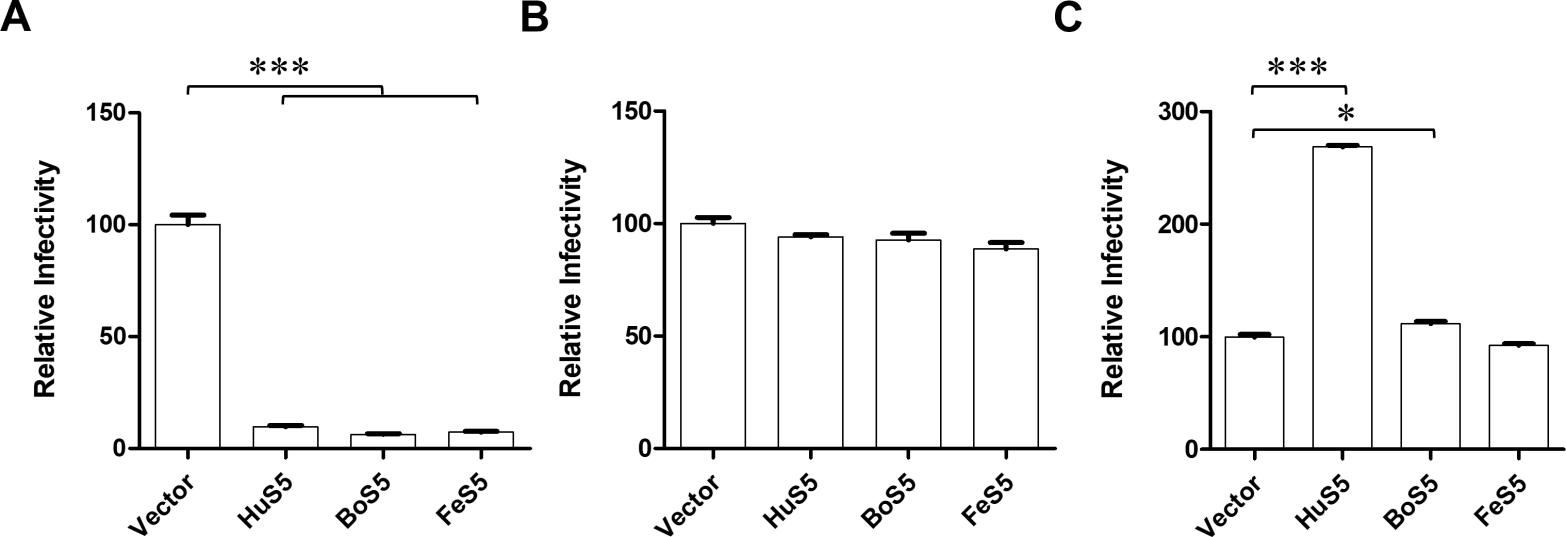
Effects of SER5 on HIV-1 Env/EBOV GP-mediated pseudovirus infection. (**A**) HIV-1 Env-mediated pseudoviral infection was assessed under SER5 expression in the cell-free mode. The statistical analysis was performed via one-way ANOVA analysis. *P* < 0.05 was considered as significant. ^⁎⁎⁎^*P* < 0.0001. (**B**) HIV-1 Env-mediated pseudoviral infection was assessed under SER5 expression in the cell-cell manner. (**C**) EBOV-GP-mediated pseudoviral infection was evaluated under SER5 expression in the cell-free manner. The statistical analysis was performed via paired two-tailed Student’s *t*-test. *P* < 0.05 was considered as significant. ^⁎^*P* < 0.05 and ^⁎⁎⁎^*P* < 0.0001.

Relying on the above investigation, we then detected whether BoS5 and FeS5 could impair viral infection *in vitro*. We first examined their anti-HIV-1 infection via the cell-free model. In the presence or absence of SER5, virion-containing supernatants were collected to infect the indicator TZM-Bl cells. As shown in Fig. 3A, compared with the control, SER5 of these mammalian species greatly suppressed HIV-1 pseudoviral infection, indicating their potent antiviral effects. Interestingly, these SER5s displayed distinct effects toward EBOV GP-mediated pseudoviral infection. In contrast to their anti-HIV-1 potency, HuS5 and BoS5 enhanced EBOV GP-mediated pseudoviral infectivity (Fig. 3C). Based on these results, we then further examined whether SER5 could block HIV-1 cell-cell infection. In this investigation, we used the HIV-1-ΔGag vector (pNL-ΔGag) as the backbone, which was defective in viral core production but retained the intact Env to mediate viral envelope-cell plasma membrane fusion. The pNL-ΔGag and SER5 expression vectors were co-transfected into 293T cells, which were digested and added to the indicator TZM-bl cells. In Fig. 3B, in the presence or absence of SER5, pNL-ΔGag nearly equally mediated the TZM-bl infection, demonstrating these SER5s lost HIV-1 Env restriction under the cell-cell infection model.

### SER5 was unable to impair BLV/BLV Env-mediated cell-cell infection

**Fig 4:**
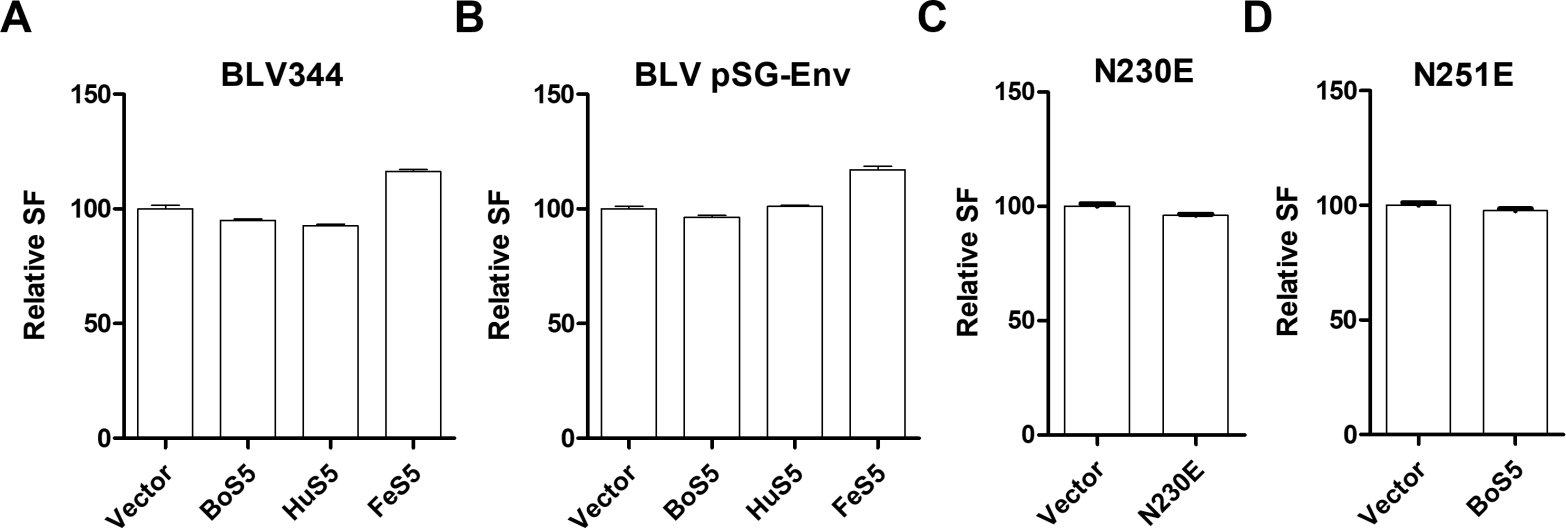
Effects of SER5 on BLV cell-cell infection. (**A**) BLV provirus clone-BLV344-mediated cell-cell SF was monitored under SER5 expression. (**B**) BLV Env (pSG-env)-mediated cell-cell SF was monitored under SER5 expression. (**C and D**) The BLV Env glycosylation variants N230E and N251E mediated cell-cell SF which was monitored under expression of BoS5. SF, syncytium formation.

BLV was known to strictly rely on the cell-cell transmission *in vivo*. Here, we tested whether SER5 could restrict BLV on its cell-cell infection. The provirus clone BLV344 (or its envelope expression vector pSG-Env) was co-transfected with different SER5s into 293T cells, which were then digested and incubated with feline kidney indicator cells—F81. BLV Env could induce F81 SF, by which we here applied to define BLV infectivity. As shown in Fig. 4A and B, BLV344/pSG-Env induced similar numbers of SF in the presence and absence of SER5s. Furthermore, BLV Envs bearing one glycosylation variation, including the N230E and N251E, were used to examine their SF induction potential in the presence of BoS5. In Fig. 4C and D, compared with the vector control, in the presence of BS5, the Env glycosylation variants induced similar SF numbers. Therefore, these results indicated SER5 did not restrict BLV cell-cell infection *in vitro*.

## DISCUSSION

SER5 was reported to potently restrict retroviral infection (10). Current studies indicated that SER5 of different species showed a highly conserved feature in their antiviral function (19). In this study, we extended to investigate BoS5 and FeS5 antiviral potency. Our results verified HuS5, BoS5, and FeS5 effectively restricted HIV-1 cell-free infection (Fig. 3A), supporting a conserved antiviral feature of SER5 across mammalian species. Furthermore, both BoS5 and FeS5 were located at the cell membrane (Fig. 2A) and capable of being incorporated into virions (Fig. 2B), as human SER5 did, demonstrating incorporation into virions was necessary for SER5 antiviral activity.

Viruses could use two transmission patterns, i.e., the cell free and cell-cell to complete their infection cycles. Host antiviral factors showed distinct effects toward viral cell-free and cell-cell infections, such as BST-2 and IFITM (2, 20, 21). Currently, SER5s antiviral studies were mainly focused on viral cell-free transmission and lack of exploration on viral cell-cell infection. Our results demonstrated that SER5s potently restricted HIV-1 cell-free infection but nearly lost the blocking under the cell-cell infection (Fig. 3A and B). It was indicated that SER5 did not directly colocalize with HIV-1 Env but probably disturbed Env clustering on the viral surface (22, 23), which was critical for HIV-1 to initiate the next round of infection. Thus, we hypothesize that under the cell-cell infection, HIV-1 Env clustering may not be susceptible to SER5 interference. Anyhow, more work is needed to clarify this mechanism. HIV-1 Nef could regulate a group of host factors other than SER5. The cell-cell transmission enabled HIV-1 to evade SER5 restriction and thus helped Nef better neutralize other host factors’ restriction. Previous reports showed that EBOV GP-mediated cell-free infection was enhanced by HuS5 (24). Here, our results also confirmed HuS5 and BoS5 enhanced EBOV GP pseudotyped viral cell-free infection (Fig. 3C). The underlying mechanism is unclear and deserves further dissection.

BLV was believed to depend on cell-cell transmission *in vivo*. In this report, we found that BLV was unrestricted by SER5 in its cell-cell infection (Fig. 4A; Fig. S1). Possibly, BLV Env was insensitive to SER5-mediated restriction (Fig. 4B). In previous reports, it was observed that some HIV-1 and EIAV envelope glycoproteins were insensitive to human and equine SER5-mediated antiviral effects (9, 25, 26). Therefore, BLV may circumvent SER5 restriction via a similar mechanism.

Envelope glycosylation modification was critical for viral infection. Several BLV envelope glycosylation sites were determined to affect its virulence (27). In this report, we used two BLV envelope glycosylation variants, both of which contained only one glycosylation substitution. Our results indicated envelope glycosylation seemed to impose less effects on BLV cell-cell transmission under BoS5 interference (Fig. 4C and D). Whether other BLV envelope glycosylation variants were insensitive to SER5-mediated restriction needs further investigation. Currently, it is still unclear whether BLV is sensitive to SER5-mediated restriction under cell-free infection. We tried to establish a BLV cell-free infection system but failed. Anyhow, further work is needed to examine this case. In addition, many genotypes of BLV have been identified (28–31). Whether these genotypes are insensitive to SER5-mediated restriction needs to be clarified.

Taken together, we tested the antivial potency of SER5 to HIV-1 and BLV under their cell-cell infection, in a scenario where SER5 lost the restriction. These results provided evidence that viruses could use cell-cell infection to evade the pressure imposed by the host innate immunity. Whether other viruses can employ the cell-cell infection to circumvent SER5-mediated restriction as HIV-1 and BLV did needs further examination.

## Data Availability

The authors confirm that the data supporting this study are available within the article.

## SUPPLEMENTAL MATERIAL

Fig. S1 spectrum.02748-24-s0001.tif BLV-mediated SF under microscopy assay.

Supplemental material spectrum.02748-24-s0002.docx Caption for Fig. S1.

ASM does not own the copyrights to Supplemental Material that may be linked to, or accessed through, an article. The authors have granted ASM a non-exclusive, world-wide license to publish the Supplemental Material files. Please contact the corresponding author directly for reuse.
