# Supplementary figures and images for: HIV-1 and BLV are insensitive to SERINC5 restriction under the cell-cell infection
Source: Microbiol Spectr. 2025 Jan 27;13(3):e02748-24. doi: 10.1128/spectrum.02748-24 (PMC11878069; doi:10.1128/spectrum.02748-24)

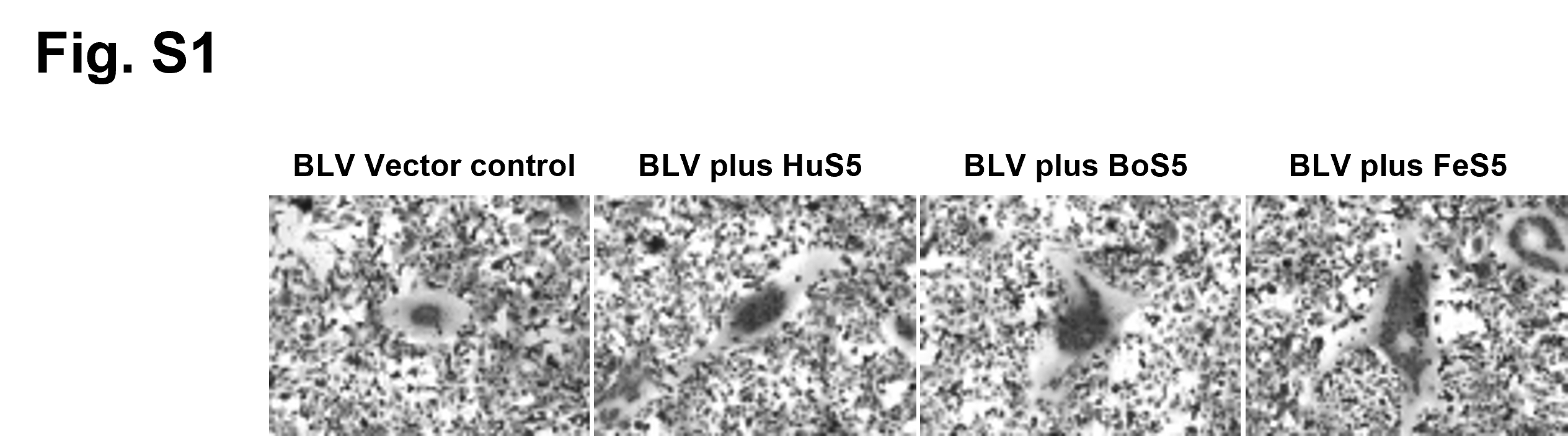

Supplement: Fig. S1 — BLV-mediated SF under microscopy assay. [file spectrum.02748-24-s0001.tif]
