# Supplementary material for: HIV-1 and BLV are insensitive to SERINC5 restriction under the cell-cell infection
Source: Microbiol Spectr. 2025 Jan 27;13(3):e02748-24. doi: 10.1128/spectrum.02748-24 (PMC11878069; doi:10.1128/spectrum.02748-24)
Supplement: Supplemental material — Caption for Fig. S1. [file spectrum.02748-24-s0002.docx]

**SUPPLEMENTARY DATA**

**Fig. S1.** BLV-mediated SF under microscopy assay. BLV344 was co-transfected with the control vector or the SER5 expression vector in 293T cells. After 12 h, 293T cells were digested and added to the F81 indicator cells. One day later, SF was pictured and numerated under microscopy. SF, syncytium formation.
